# Using Cognitive Agents to Train Negotiation Skills

**DOI:** 10.3389/fpsyg.2018.00154

**Published:** 2018-02-19

**Authors:** Christopher A. Stevens, Jeroen Daamen, Emma Gaudrain, Tom Renkema, Jakob Dirk Top, Fokie Cnossen, Niels A. Taatgen

**Affiliations:** Faculty of Science and Engineering, Artificial Intelligence and Cognitive Engineering, University of Groningen, Groningen, Netherlands

**Keywords:** theory-of-mind, negotiation, cognitive modeling, strategic games, training

## Abstract

Training negotiation is difficult because it is a complex, dynamic activity that involves multiple parties. It is often not clear how to create situations in which students can practice negotiation or how to measure students' progress. Some have begun to address these issues by creating artificial software agents with which students can train. These agents have the advantage that they can be “reset,” and played against multiple times. This allows students to learn from their mistakes and try different strategies. However, these agents are often based on normative theories of how negotiators should conduct themselves, not necessarily how people actually behave in negotiations. Here, we take a step toward addressing this gap by developing an agent grounded in a cognitive architecture, ACT-R. This agent contains a model of theory-of-mind, the ability of humans to reason about the mental states of others. It uses this model to try to infer the strategy of the opponent and respond accordingly. In a series of experiments, we show that this agent replicates some aspects of human performance, is plausible to human negotiators, and can lead to learning gains in a small-scale negotiation task.

## Introduction

Negotiation is an important tool through which people work with others to better satisfy their needs. Negotiation is ubiquitous, and its contexts range from mundane daily occurrences (e.g., deciding how to split the check for dinner) to historic, far-reaching events (e.g., international conflict resolutions). For this reason, it is important for people to know how to effectively approach negotiations in order to achieve fair, mutually beneficial agreements. However, training negotiation is challenging because it is a complex activity that involves at least two parties. So people must practice either in groups or with simulated partners. Cognitive agents are a promising tool for developing such agents because they can simulate human memory, biases, and problem solving strategies, allowing students to get a better sense of how real negotiators will respond to various circumstances. Here we develop and validate a cognitive agent that can perform a single-issue bargaining task.

### Cognitive agents as training partners

Prior work shows that cognitive agents can make realistic opponents and training partners in multi-person tasks. These agents provide a good account of behavior in a variety of multiplayer strategic games including the Prisoner's Dilemma (Gonzalez et al., 2014), Rock-Paper-Scissors (West et al., 2005), and even Backgammon (Sanner et al., 2000). Moreover, in complex cooperative tasks such as UAV piloting, teams with a cognitive agent can perform just as well as all-human teams (Ball et al., 2009). However, there is a dearth of research on the effect of these agents on learning. We aimed to address this gap in the current work by demonstrating the utility of a cognitive agent for negotiation training.

### Theory of mind in negotiation

Prior research suggests that theory of mind, the ability to reason about the beliefs and intentions of others, may play an important role in negotiation (Hindriks and Tykhonov, 2008; de Weerd et al., 2013, 2015a). People don't always use theory of mind when it is to their advantage (Hedden and Zhang, 2002), but training with software agents that have theory-of-mind capabilities can improve theory-of-mind utilization in human users (de Weerd et al., 2015a). In the present paper, we develop a prototype cognitive model with theory-of-mind capabilities to serve as a training partner for humans learning to negotiate.

Fisher and Ury (1981), admonish negotiators to focus on interests, not positions. In other words, a skillful negotiator bases decisions on his/her own goals and the goals of the opponent(s). Unfortunately, in negotiations the true goals of the opponent are often unclear. Making inferences about these unknown goals requires theory of mind (Premack and Woodruff, 1978), the ability to reason about the mental states of oneself and others.

Theory of mind provides an important advantage in a variety of settings. Agents that possess theory of mind often outperform their opponents in competitive settings (Hindriks and Tykhonov, 2008; de Weerd et al., 2014), and achieve better outcomes for themselves and their teammates in cooperative and mixed-motive situations, including negotiation games (de Weerd et al., 2013; Stevens et al., 2016). In a mixed-motive situation, the goals of the players partially overlap, making theory of mind especially helpful in identifying areas of common interest (de Weerd et al., 2015b). Unfortunately, there is compelling evidence that people often do not use theory of mind when it is useful to do so (Hedden and Zhang, 2002; Wright and Leyton-Brown, 2010; Camerer et al., 2015).

It remains an open question how best to train theory of mind in negotiators. Training with software agents appears to be a promising option. de Weerd et al. (2015b) show that people do show evidence of adopting theory of mind after negotiating with an agent with theory of mind. Unfortunately, people often behave differently when dealing with agents than when dealing with people (Kiesler et al., 1996; Lin et al., 2014), making it possible that people will be reluctant to ascribe mental states to agents. Thus, an ideal software agent is one that makes decisions in a realistic and plausible way. In the present study we create such an agent by utilizing Instance-Based Learning (Logan, 1988; Gonzalez and Lebiere, 2005), a theory that has been successful in modeling human decision-making in a variety of contexts, including the prisoner's dilemma (Lebiere et al., 2000; Gonzalez et al., 2014; Stevens et al., 2016), backgammon (Sanner et al., 2000), and the lemonade game (Reitter et al., 2010).

### Cooperative and competitive goals in negotiation

A key challenge in negotiation is balancing cooperation and competition. Negotiators must work together to create valuable agreements, but they must also claim some of the created value for themselves (a.k.a. “The Negotiator's Dilemma”; Lax and Sebenius, 1986). Almost all negotiations are mixed-motive in the sense that failing to reach an agreement yields no benefit. So the goals of the other party must (at least to a degree) be satisfied. However, people may differ in the relative weights they assign to their own goals and to the goals of others (De Dreu et al., 2000). Competitive players have a low concern for the outcomes of others while cooperative players have a high concern.

Depending on intentions, negotiators will use different types of strategies (Allen et al., 1990; Huffmeier et al., 2014). Cooperative strategies aim to maximize the probability of an agreement and the satisfaction of all parties involved, and thus are very appropriate if one's goal is to cooperate. These strategies are characterized by moderate opening bids, high reciprocity, willingness to make unilateral concessions, honesty, and open sharing of information (Yukl, 1974; Esser and Komorita, 1975; Komorita and Esser, 1975; Allen et al., 1990; Paese and Gilin, 2000; Huffmeier et al., 2014). The philosophy behind these strategies is summarized by Osgood's Graduated Reciprocation in Tension (GRIT) Theory (Osgood, 1962). According to this theory, offering an opponent a concession can reduce the tension felt by the other party, increasing the probability of a concession in return.

Aggressive strategies, by contrast, are intended to maximize one's own gains without regard to the gains of the other negotiator(s). In some cases, those using aggressive tactics may actually want their counterpart to receive as little value as possible from the deal (Aksoy and Weesie, 2012). Aggressive strategies are characterized by high opening bids, low reciprocity, unwillingness to make unilateral concessions, deception, and application of time pressure (Siegel and Fouraker, 1960; Chertkoff and Baird, 1971; Esser and Komorita, 1975; Smith et al., 1982; Allen et al., 1990; Huffmeier et al., 2014). These strategies are based on aspiration theory (Siegel and Fouraker, 1960). Openly aggressive moves communicate to the opponent that the bargainer's goal is to maximize his or her own payout. According to aspiration theory, when a negotiator has high aspirations, the opposing negotiator will tend to lower his/her aspirations in response. Aspiration theory prescribes that bargainers always show strength in negotiations, as that will weaken the aspiration levels of the opponent. Aggressive negotiators might also engage in deception in order to hide their aggression and exploit the trust of a counterpart (Chertkoff and Baird, 1971). This deception can include lying about reserve prices, alternative offers, the value of an item for sale (in the case of a buyer-seller negotiation), etc.

Knowing the intentions of the opponent can provide an important advantage by allowing the player to adapt their own strategy accordingly. In mixed-motive contexts, the best way to adapt is often to become as cooperative (or aggressive) as the opponent, a meta-strategy known as matching (Liebert et al., 1960; Druckman et al., 1972; Yukl, 1974; Chertkoff and Esser, 1976; Smith et al., 1982; Faratin et al., 1997; Maaravi et al., 2014). This type of meta-strategy is commonly observed in social interaction games, such as the ultimatum game (Falk et al., 2003; Falk and Fischbacher, 2006) and the prisoner's dilemma (Kelley and Stahelski, 1970; Stevens et al., 2016). It has also been observed in negotiation experiments, especially when participants have more information about their counterparts' payoff schemes and alternatives (Liebert et al., 1960; Smith et al., 1982; Weingart et al., 2007; Schei et al., 2011). This meta-strategy is reminiscent of the tit-for-tat strategy in the prisoner's dilemma (Axelrod, 1980; Lax and Sebenius, 1986). A matching meta-strategy rewards cooperative behavior by an opponent and punishes aggressive behavior. Previous work has shown that negotiators will sometimes employ a matching meta-strategy. However, the lack of time and information in many negotiation contexts makes this difficult (Liebert et al., 1960; Chertkoff and Esser, 1976; Smith et al., 1982).

### The game of nines

The focus of the present work is on single-issue, distributive negotiation: two parties negotiating to determine how to divide a fixed amount of value. Our experimental task is the Game of Nines, a negotiation task first used by Kelley et al. (1967) to study negotiation behaviors in human participants. In this game, two players are given 9 points that must be split between them. In addition, each player has a minimum value that they must acquire in order to avoid losing points (Minimum Necessary Share, or MNS). When a player receives points, their MNS is subtracted from these points to determine their profit or loss. For instance, if a player's MNS is 2, and they receive 3 points, their profit for the round is 1. Each player knows only their own MNS value, but not their opponent's. If the players cannot reach an agreement, neither player gets any points. The game is played over a series of rounds, each requiring the players to divide nine points. The structure of the negotiations is often open-ended, allowing the players to discuss potential deals as they wish.

Like many real-world negotiations, the Game of Nines is a mixed-motive situation. Players are motivated to work for the interests of the group but they also have interests that conflict with those of the group. Both players have a clear incentive to reach an agreement because that is the only way to obtain points. However, players are also motivated to receive as many points as possible out of every deal. Increasing one's own points can only be done at the expense of the other player.

## Overview of experiments

Our goal in this paper is to produce a cognitively plausible negotiation training agent. We conducted three experiments to this end. In Experiment 1, we observed how people negotiate with two non-cognitive agents. We then used this data to build a cognitively plausible agent that can play the Game of Nines. We verified the plausibility of this agent by pitting it against non-cognitive agents in the Game of Nines. In Experiment 2, the model played against human players so that we could determine its effectiveness and believability. In Experiment 3, we used the model to train human participants in the Game of Nines. Experiment 1 showed that the model negotiates in a similar way as humans in this task. Experiments 2 and 3 showed that the model is a competent, believable negotiation partner and that people who play against it show improvement in the Game of Nines.

## Experiment 1

In Experiment 1, human participants played the Game of Nines against two pre-programmed, non-cognitive agents. The two artificial agents used different strategies; one was more cooperative, while the other was more aggressive. To perform well in the task, players should adapt their strategies to counter those of their opponent. We then examined the strategies and performance of the human players and used this data to construct a metacognitive model capable of identifying and adapting to these strategies. Finally, we allowed our metacognitive model to play against the non-cognitive agents to determine whether it demonstrated human-like performance.

### Participants

Twenty-one people (8 male, age *M* = 21.8 *SD* = 2.1) participated in this experiment in exchange for 10 euros. This group consisted mainly of international students at the University of Groningen. All were recruited using ads posted on social media.

### Procedure

Participants in all three experiments completed the experimental task in a small laboratory equipped with 3 cubicles, each containing a Macbook Pro laptop computer. Each participant was seated in one of the three cubicles. Participants in Experiments 1 and 3 interacted with software agents loaded onto their respective laptops. Participants in Experiment 2 interacted with the experimenter and a confederate through a local wireless network connecting the laptops in the cubicles.

We used two non-cognitive agents as opponents in this task. One opponent, the “fair” agent, was designed to play cooperatively and always attempt to achieve an even split. The other opponent, the “unfair” agent, was programmed to play aggressively and try to obtain a larger share of the points for itself. All participants played against both agents in alternating order. Half of the participants played against the fair agent first and the other half played against the unfair agent first. Participants played for 3 blocks of 24 trials per block. For half of each block, participants played against one of the agents, and then for the second half they played the other agent (order was randomly determined for each participant but consistent across the three blocks). There was a brief rest period between blocks. Each agent had a different name (“Tom” and “Ben”) and a different line-drawing for their portrait. The players were not informed about the nature of the strategies used by the agents or even that there was a difference in their strategies. The names and portraits were selected so neither would provide any clues about the nature of the agent and that each agent had the same “gender.” This was intended to prevent the participants from behaving differently toward the agents on account of gender biases.

**Figure 1:**
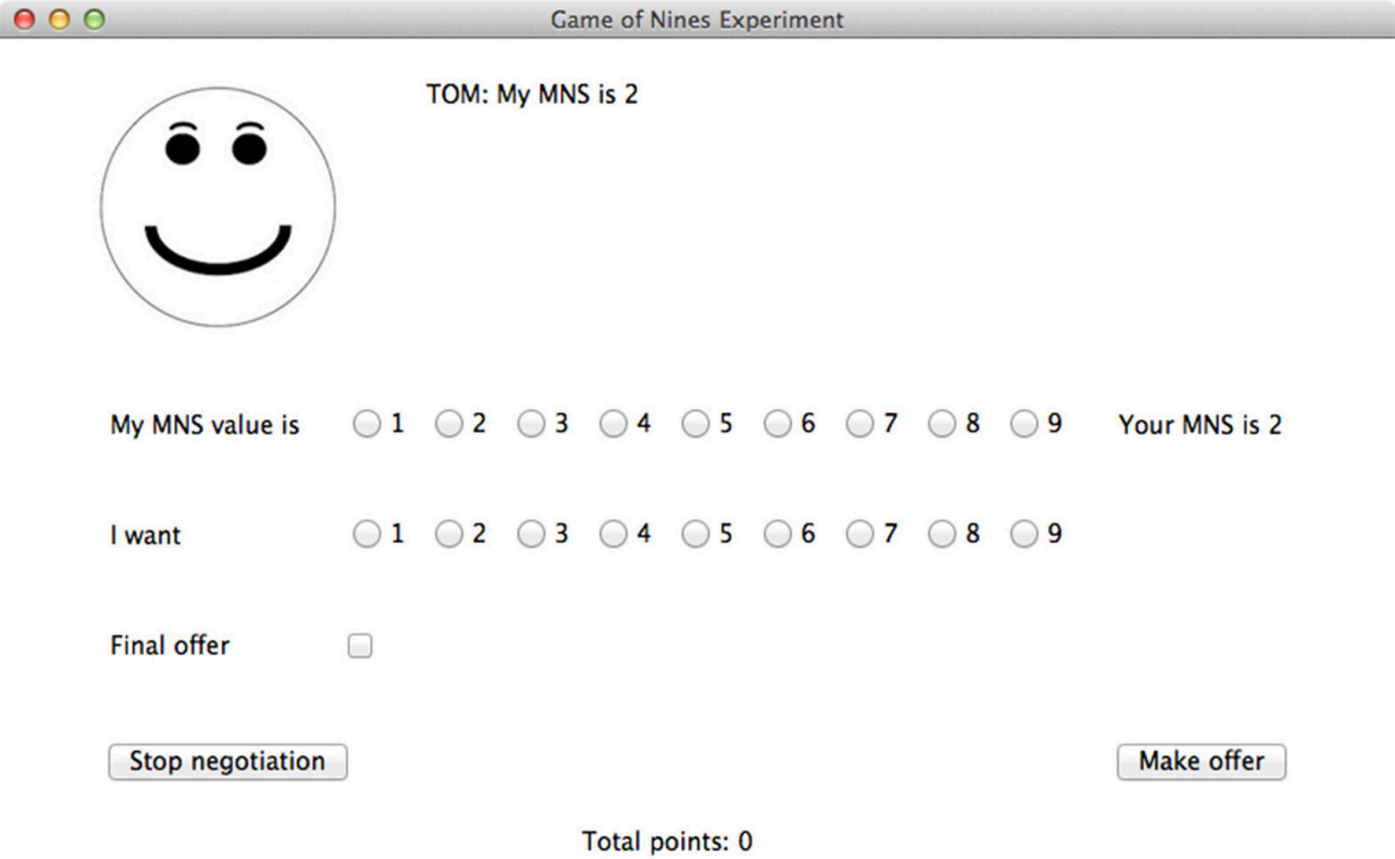
The user interface for the Game of Nines experiment.

Participants interacted with the agent through a GUI run in Python 3.3 using the TkInter library (see Figure 1). At the beginning of every trial, both the player and the agent were shown their own MNS values. The following pairs of MNS values were used: (2,2) (1,3) (3,1) (2,2) (3,3) (2,3) (3,2) (3,4) (4,3) (2,4) (4,2) (4,4). For all of these pairs, it is possible to find at least one solution in which neither party loses points, and in all but one case (4,4), it is possible for both parties to gain points. The order of these pairs was randomized for each participant and for each block. After the agent and player saw their MNS values, they were required to declare their MNS values. These declared values were visible to both the player and the agent and did not have to be true. Both the agent and the player could display dishonest MNS values. Each player knew only their own MNS value and their opponent's declared MNS value. Then, the player was asked to make an offer to the agent. This offer indicated how many points the player wanted for himself or herself. Any number from 1 to 9 could be selected. The player also had the option to indicate that this was his or her final offer. This indicated to the agent that the player would not make any lower offers. Finally, the player could quit the negotiation instead of making an offer. After the player made an offer, the agent would accept it, propose a counter offer, or quit. This process continued until an agreement was reached or one player quit. At the end of the trial, the player was notified of the outcome of the trial and the player's cumulative number of points for the block was displayed. The player was not informed about how many points the agent had scored.

### Non-cognitive agents

The non-cognitive agents negotiate based on simple algorithms and are not based on models of human reasoning or memory. These models also do not possess theory of mind capability. The non-cognitive agents were designed to play either cooperatively or aggressively. However, they each respond better to a different type of negotiation strategy. To perform well in this task, a player cannot simply use the same strategy against both agents. The player needs to detect the strategy used by the agent and adjust his or her own strategy accordingly.

#### Fair agent

The fair agent uses a simple cooperative strategy. The agent determines the most fair point split based on its own MNS value and the assumed MNS value of the opponent. It then makes bids that are the same distance away from this split as the opponent's actual bid. However, if the agent detects a discrepancy between the player's average MNS claims and its own average MNS values, it becomes less generous. The larger the difference between the agent's mean MNS and the average reported MNS of the player, the fewer points the model will agree to grant to the player. See Appendix B in Supplementary Material for more detail on the implementation of the agents.

The ideal strategy against this agent is honest cooperation. If the player always honestly informs the fair agent about its MNS value then the agent will agree to split the available points. However, lying is very costly, as it will cause the agent to become demanding very quickly.

#### Unfair agent

The unfair agent, by contrast, was designed to maximize its own profit. The agent routinely lies about its own MNS value and makes high demands of the player. Like the fair agent, this agent tracks the player's honesty but it is equally demanding regardless of how honest the player is.

One final difference between the two agents is how they handle a player's final offer. The fair agent has a small chance of accepting an offer lower than its fair offer. But it will not accept anything 3 or more points less than the fair offer. When the unfair agent receives a final offer, it considers how many points it will gain. This agent has a high probability of accepting a final offer as long as it will make a profit of at least one point. Thus, the use of the final offer option is much more effective against this agent.

### Results

A linear mixed effects model (Bates et al., 2014; Kuznetsova et al., 2015) with trial block and agent type as fixed factors and subject as a random effect was fitted to the data and the fixed effects parameters were tested for significance with *t*-tests. For binomially distributed variables (final offers and agreements), a generalized linear mixed effects model was fitted instead. Model estimates, as well as their standard errors and *t*-values, are reported in Tables 1–3.

**Table 1:** LME analysis on points earned.

	**Estimate**	**SE**	***df***	***t***	***p***
Block	1.05	0.61	102	1.72	0.09
Agent type	−0.02	1.86	102	−0.01	0.99
Block × Agent type	0.12	0.86	102	0.14	0.89

*Agent Type was dummy coded (0 = Fair Agent; 1 = Unfair)*.

**Table 2:** Results of LME analysis on exaggeration of MNS values.

	**Estimate**	**SE**	***df***	***t***	***p***
Block	0.02	0.06	1,488	0.35	0.73
Agent type	0.21	0.19	1,488	1.09	0.28
Block × Agent type	−0.07	0.09	1,488	−0.83	0.41

*This DV is computed by subtracting the player's actual MNS value from the value they report to the agent. Agent Type was dummy coded (0 = Fair Agent; 1 = Unfair)*.

**Table 3:** Results of GLME analysis on use of final offers.

	**Estimate**	**SE**	***Z***	***p***
Block	0.25	0.11	2.33	0.02^*^
Agent type	1.02	0.33	3.11	<0.01^**^
Block × Agent Type	−0.16	0.15	−1.07	0.29

*This DV is defined as the proportion of trials on which the participant made a final offer*.

* *, significant at p < 0.05*,

** *, significant at p < 0.01. Agent Type was dummy coded (0 = Fair Agent; 1 = Unfair)*.

#### Strategic adaptation

Overall, there was no significant difference between subjects' performance against the fair and unfair agents. (*M*_fair_ = 12.2 points, SE = 0.6; *M*_unfair_ = 12.5 points, SE = 0.9). There was some evidence of strategic adaptation based on the agent. The participants learned to use the final offer move more often against the unfair agent (*M*_fair_ = 51%, SE = 7%; *M*_unfair_ = 63% points, SE = 6%) (*Z* = 3.114, *p* < 0.002), and they used the final offer option more often as the experiment progressed, but there was no interaction between these two factors, meaning that this strategic adjustment did not change with practice. Further, participants exaggerated their MNS values by about the same amount regardless of the agent (*M*_fair_ = 1.17, SE = 0.13; *M*_unfair_ = 1.24, SE = 0.11). Finally, there was a numerical trend such that subjects were more successful in reaching agreements with the unfair agent than with the fair agent (*M*_fair_ = 60%, SE = 4%; *M*_unfair_ = 75% points, SE = 3%), but this effect did not reach significance (*Z* = 1.07, n.s.).

#### Learning

**Figure 2:**
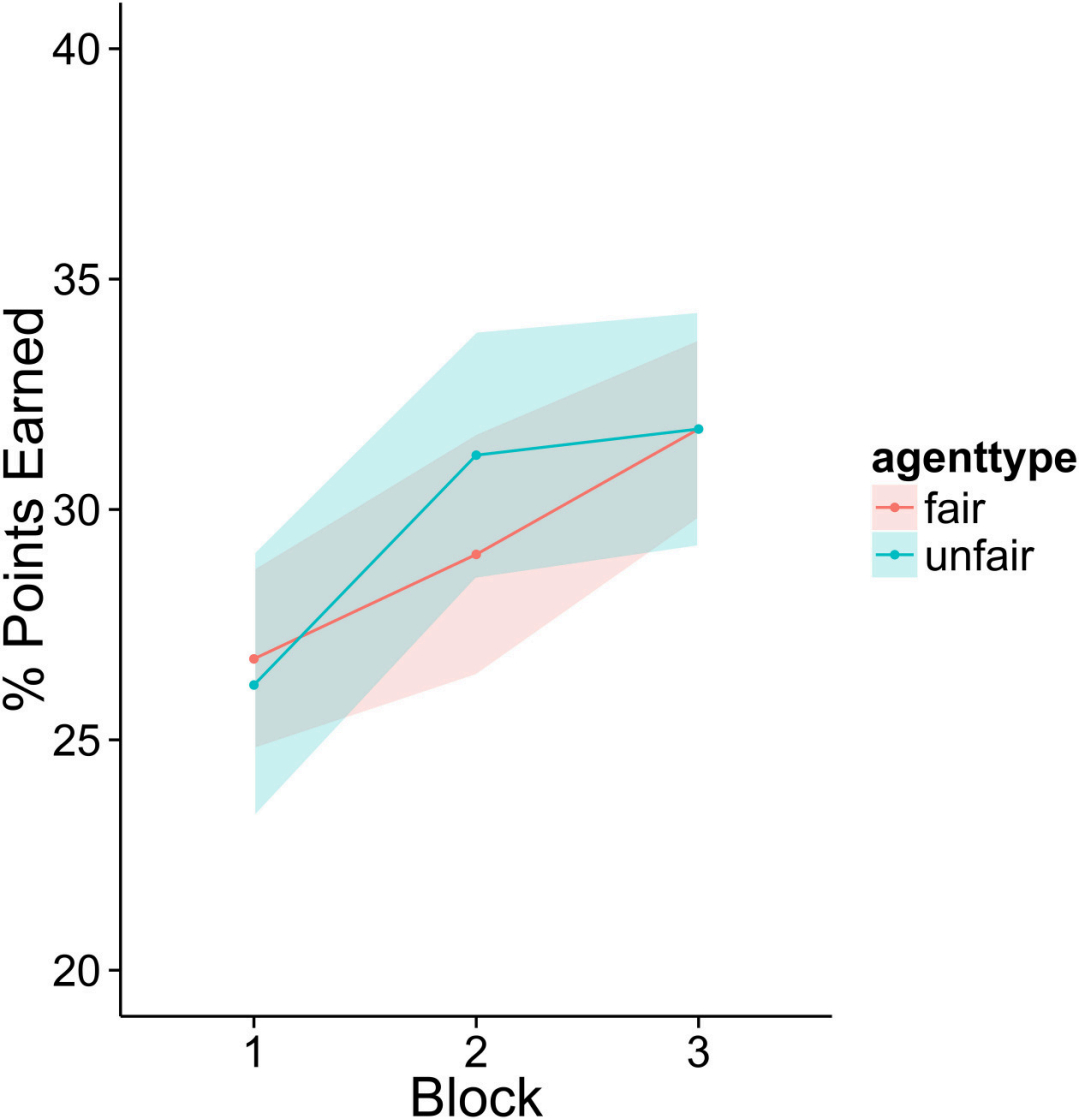
The percentage of total points available in each block earned by the participants. There was very little change across the three blocks of the experiment. Fifty percent represents the ideal outcome: the agent and player each take half of the possible points. None are wasted due to failures to reach agreement.

Participants in this experiment did not appear to improve with practice in a meaningful way (see Figure 2). Analyses on lying and final offers showed no evidence of a strategy shift over the course of the experiment (there were no significant interactions between agent type and block). There was a small trend such that participants scored more points as the experiment progressed, but this effect did not reach significance [Estimate = 1.05, SE = 0.61, *t*_(102)_ = 1.7, *p* = 0.09]. The numerical magnitude of this trend was also very small, average performance in block 3 was only 2 points higher than block one.

**Figure 3:**
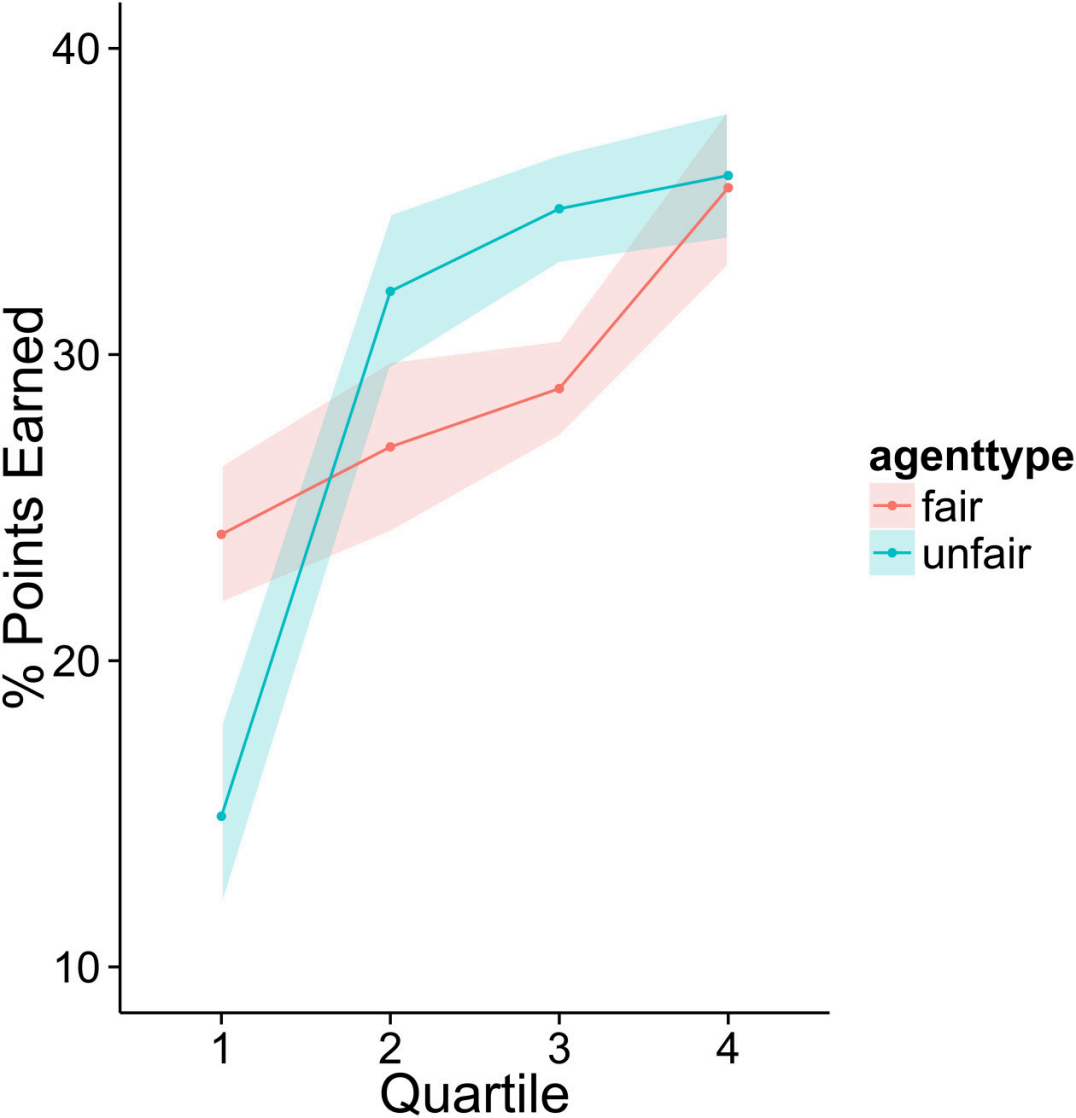
Average number of points scored against each agent by members of each quartile.

**Figure 4:**
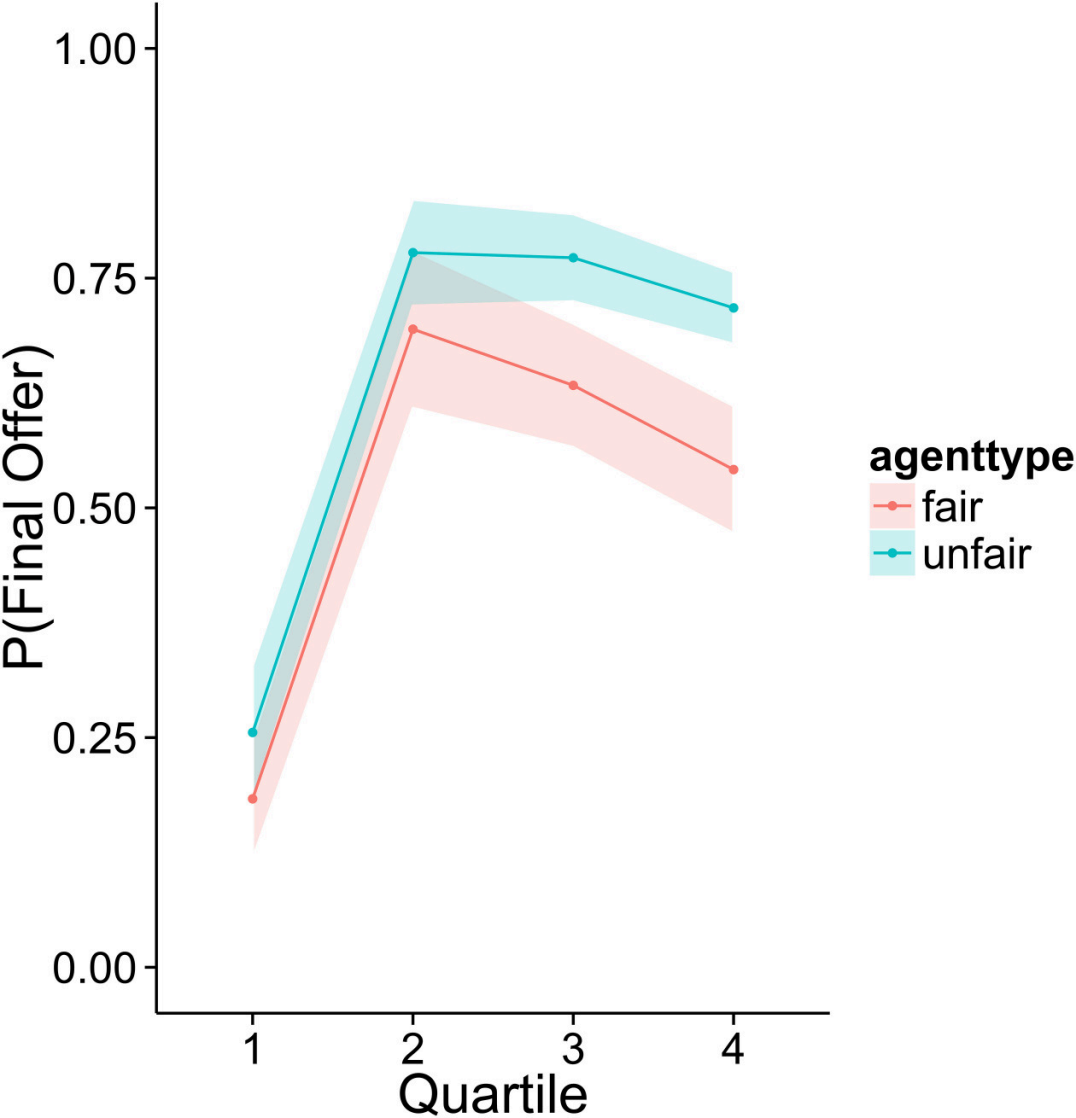
Average probability of using the final offer option as a function of agent and quartile.

**Figure 5:**
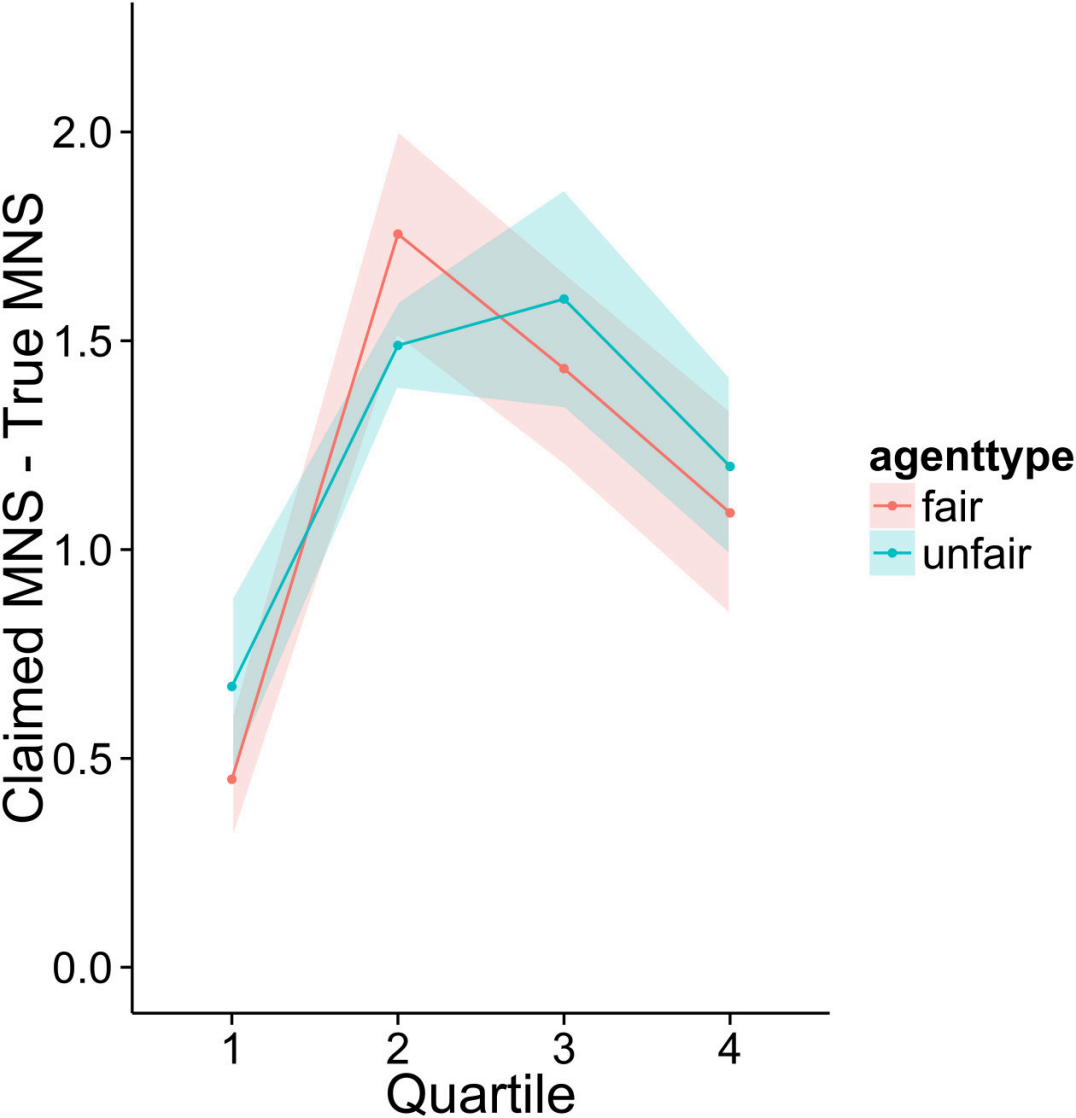
Difference between claimed and actual MNS values as a function of quartile and agent type.

**Figure 6:**
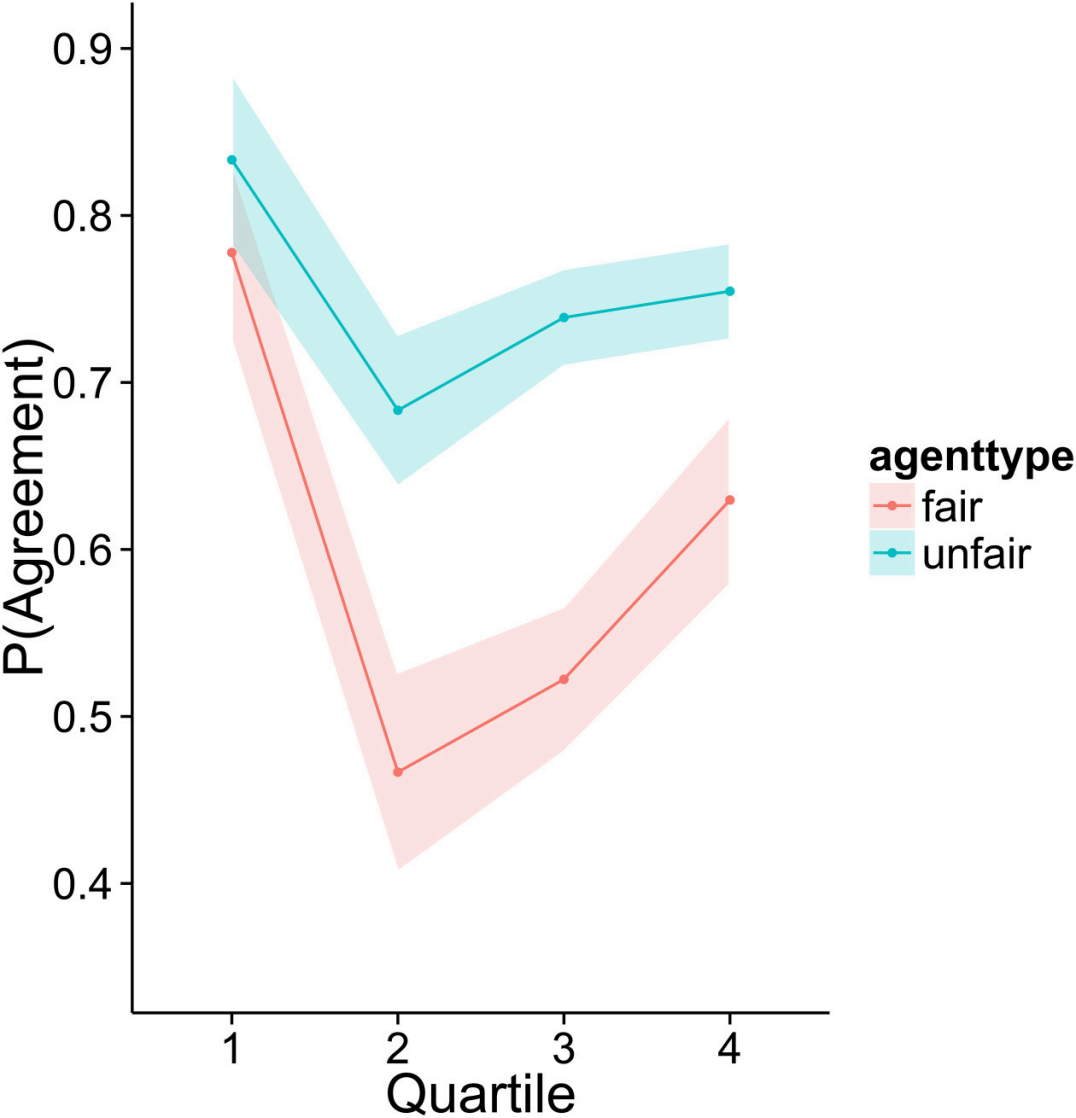
Proportion of trials in which an agreement was reached.

To investigate how higher performing participants' strategies differed from lower performers, we divided the sample into quartiles based on overall score. Figures 3–6 display the data by quartile. Quartiles were defined by a subject's overall score for the entire experiment. Overall, dividing the data in this way suggests that the strategic adaptation we observed is primarily driven by the top quartile of the sample. Participants in the middle quartiles performed better against the unfair agent than the fair agent, and participants in the bottom quartile performed poorly against both agents (see Figure 3). The top-quartile subjects have learned that making final offers works better against the more aggressive unfair agent than against the fair agent (see Figure 4). Further, subjects in the upper 2 quartiles appear to also be sensitive to effects of their honesty on the fair agent's behavior. These subjects exaggerate their MNS values to a lesser extent when playing against the fair agent (see Figure 5). These differences in strategy are reflected in overall scores and rates of agreement. Participants in the top quartile play well against the fair and unfair agents. However, participants in the lower quartiles play noticeably worse against the fair agent. Finally, participants in the bottom quartile appear to be using an overly generous cooperative strategy, as evidenced by their high rate of agreement (see Figure 6) while still receiving a low score.

## The metacognitive model

We developed a model that can perform the Game of Nines task in the ACT-R cognitive architecture (Anderson et al., 2004). This model is capable of identifying the strategy type of the opponent (cooperative or aggressive) and adjusting its own behavior accordingly. This model possesses multiple strategies in its declarative memory. As the negotiation progresses, the model infers the type of opponent it is playing by comparing the opponent's behavior to the behavior predicted by these strategies. Once it has classified its opponent, the model will select the strategy it deems most appropriate for the given opponent.

### Declarative memory in ACT-R

The primary theoretical mechanism driving our agent's behavior is ACT-R's declarative memory system. Previous work shows the dynamics of this system provide a good explanation of behavior in strategic games (West et al., 2005; Gonzalez et al., 2014). Our model contains a set of instances or cases that describe possible moves and the MNS values and strategies associated with those moves. The instances are stored as chunks in ACT-R's declarative memory. A chunk is a schematic unit of information that has one or more slots containing values or links to other chunks in declarative memory. Each chunk also possesses an activation value. More active chunks are more likely to be retrieved in a search of declarative memory. The activation level of a chunk (i) is derived from the following equation:

$$A_{i}=B_{i}+P_{i}+\;Logistic\left(0,\;s\right)$$

In this equation, B_*i*_ refers to the base level activation of the chunk. All chunks had a base level activation of 0.0 that did not change throughout the experiment. P_*i*_ is the mismatch penalty for chunk *i* (see next paragraph). The rightmost term of the equation represents noise added to the activation level. Prior research has shown that this memory noise provides a good theoretical account of variability in strategic behavior (West et al., 2005).

A chunk does not have to be a perfect match to a retrieval request in order to be retrieved. When a chunk in declarative memory is not a perfect match to a retrieval request, then ACT-R will reduce its activation according to the following formula:

$$P_{i}=\sum_{l}PM_{li}$$

*P* is the mismatch scaling factor. When *P* is higher, activation is more strongly affected by mismatching. In this model it is set to 5. *M* indicates the similarity value between the relevant slot value in the retrieval request (*l*) and the corresponding slot chunk *i* (the chunk in declarative memory) summed over all slot values in the retrieval request. For numerical values, the similarity score was computed using the following function:

$$Mli=\frac{1}{\frac{{\left(l-i\right)}^{2}}{2}+1}-1$$

This function ensures that, for any two values, *l* and *i*, the similarity score will be between −1 (most dissimilar) and 0 (least dissimilar or identical). Moreover, the similarity values between “aggressive” and “cooperative” in the strategy slots were set at −1, but the similarity between aggressive and neutral and cooperative and neutral was set to −0.1. Thus, if the system is searching for a cooperative instance, it is most likely to retrieve a cooperative instance, and it is more likely to retrieve a neutral instance than an aggressive instance. Similarities between all other non-numerical values were set to −1.

Our model uses the same instances to guide its own behavior and to interpret the behavior of others. Each instance represents a move and a context in which to make that move. Some of these instances are characteristic of cooperative players (e.g., conceding even when your offer is close to your minimum) and others are more characteristic of aggressive players (e.g., declaring that this is your last offer, even though you still have room to concede). An initial set of instances was hand-coded by the authors following the above mentioned guidelines. These instances were down-selected and adjusted manually in order to improve the fit of the agent's predictions to the data from Experiment 1. The final set of instances can be found in Appendix A in Supplementary Material. The model contains a total of 30 instances: 12 cooperative, 9 aggressive, and 9 neutral. The model chooses an action based on its own chosen strategy and context. Similarly, it decides which strategy its opponent is using based on its opponent's action and context.

#### Selecting an action

The model retrieves instances based on conditions. These conditions specify important information about the game, such as the distance between the model's current offer and its MNS value (offer-difference), the opponent's previous move (did they concede and by how much?), and the model's current selected strategy (see below for details on strategy selection). Once the conditions have been calculated by the model, ACT-R's partial matching mechanism is used to determine which instance is the best match to the current situation. The model then applies the move specified by the best matching instance. The possible moves include initial-offer, concede, insist, final-offer, and quit (based on the classification system proposed by Filzmoser and Vetschera, 2008). An initial-offer instance specifies the first offer the model should make in a given round. Concede tells the model to reduce its current offer. Insist tells the model to re-submit the current offer. Final-offer causes the model to indicate that it will not accept any less than the current offer. Finally, quit simply instructs the model to quit the round.

The instances are classified into three different strategy types: Cooperative, Aggressive, and Neutral. Cooperative instances are modeled after softline bargaining strategies (Esser and Komorita, 1975; Huffmeier et al., 2014). In general, they instruct the model to make lower opening offers and to concede frequently, especially when the model's current offer is far above the model's MNS value and when the opponent offers a concession. Aggressive instances call for higher opening offers and less frequent concessions (Siegel and Fouraker, 1960). In addition, whereas the cooperative instances will always tell the model to be honest in reporting its MNS value, aggressive instances instruct the model to exaggerate its MNS value. Finally, the model will make final offers when using the aggressive strategy, but will not when using the cooperative strategy. The model also includes neutral instances. These instances represent behavior that is ambiguous or appropriate regardless of one's goal.

#### Theory of mind

The model uses these same instances to determine the opponent's strategy. Every time the opponent makes a move, the model assumes the perspective of the opponent and attempts to determine if the move is most similar to the cooperative, aggressive, or neutral instances. The model searches declarative memory using the opponent's move, the model's previous move, and an estimate of the opponent's distance to their MNS value. If the instance retrieved is cooperative or aggressive, the model increases its confidence that the opponent is using the retrieved strategy. If it is neutral, the model ignores the move and keeps its previous estimate. The model has two additional chunks in memory, labeled cooperative and aggressive, to track the opponent's relative use of each strategy. Each time the model detects one of the two strategies it reinforces the associated chunk. To determine which strategy the opponent is using overall, it retrieves the most active of these two chunks from memory. This retrieval is governed by the same activation and noise functions described above. The implication is that the agent's belief about the strategic stance of its opponent will fluctuate based on the recency and frequency of prior evaluations, as well as system-wide noise in declarative memory. The frequency effects enable the agent to build up confidence about the opponent's strategy while the recency effects allow the model to adapt to strategy shifts.

To interpret an opponent's move, the model considers two primary factors: reciprocity and minimal goals. Reciprocity is the tendency for the opponent to concede after the model concedes. High reciprocity is a characteristic of cooperative players (Esser and Komorita, 1975; Huffmeier et al., 2014). Thus cooperative instances will tend to respond to concessions with concessions, but aggressive instances may be more likely to insist. Minimal goals refer to the smallest payout a player is willing to accept from a negotiation. This is influenced by their MNS values. Behavior that appears aggressive could simply be the result of a high minimal goal on a given round. Although the model does not know the opponent's MNS values, it knows that (as the human players did in the experiment), on average, the opponent's average MNS values will be the same as its own. Therefore, the model stores its past MNS values in declarative memory and uses these memories to estimate its opponent's minimal goal in a given round. Aggressive behaviors are defined as those having a high value relative to a player's minimal goal.

Once the model has determined the type of opponent it is playing against, it must decide how to respond. If the opponent is a cooperative player, the model will also choose a cooperative strategy, but if the opponent is aggressive, the model will choose an aggressive strategy. Once a counter-strategy is chosen, the model will use it to search for an appropriate instance in memory. See Table 4 for example instances. The chosen strategy is used as a retrieval cue when selecting an instance from memory as described above. Once the model has chosen a strategy, it is most likely to select instances from that strategy. Instances belonging to the other strategy types receive a mismatch penalty according to the formula described above. The M_*li*_ values are set as follows. The aggressive and cooperative strategies are maximally different from one another (*M*_*li*_ = −1). However, the neutral strategy is more similar to both strategies (*M*_*li*_ = −0.1). As a consequence, the model will usually pick instances from its chosen strategy, but will also sometimes select instances from the neutral strategy. Because of the high mismatch penalty, the model will rarely select instances from the opposite strategy.

**Table 4:** Example instances.

**Slot name**	**Value**	**Interpretation**
**EXAMPLE 1**		
My-strategy	Cooperative	I am using the cooperative strategy.
MNS-Bid Diff	3	My previous bid was three points higher than my MNS value.
Opponent-move-type	Concede	My opponent's previous bid is lower than the one he made before that.
Opponent-move-value	1	My-opponent's previous bid is one point lower than the one before it.
My-move-type	Concede	I will now submit a lower bid than my previous bid.
My-move-value	1	My bid will be one point lower than my previous bid.
**EXAMPLE 2**		
My-strategy	Aggressive	I am using the aggressive strategy.
MNS-Bid Diff	3	My previous bid was three points higher than my MNS value.
Opponent-move-type	Concede	My opponent's previous bid is lower than the one he made before that.
Opponent-move-value	1	My-opponent's previous bid is one point lower than the one before it.
My-move-type	Insist	I will now resubmit my current bid.
My-move-value	0	My new bid will be no different than my current bid.

## Simulation results

Three different versions of the cognitive model were played against the fair and unfair agents: cooperative, aggressive, and metacognitive. The cooperative model always used the cooperative strategy, the aggressive model always used the aggressive strategy, and the metacognitive model changed strategies depending on its opponent's behavior. Just like the human participants, the cooperative, aggressive, and metacognitive models played against the fair and unfair agents for three blocks of 12 trials each. To ensure stable data, we ran the simulation for 500 simulated subjects.

As mentioned in the behavioral results, we suspected that the top participants were using theory of mind, while the middle subjects were using an aggressive strategy. We divided the subjects into three groups based on hypothesized strategy: the first group consisted of the top quartile (*n* = 6), the second group consisted of the middle two quartiles (*n* = 10), and the third group consisted of the bottom quartile (*n* = 5). We hypothesized that the first group used theory of mind to adapt to their opponents. Therefore, we expected the metacognitive model to be the best fit. By contrast, we hypothesized the second group used an aggressive strategy, and would therefore be best fit by the aggressive model. The third group was not modeled due to a lack of clear strategy.

### Model performance

**Figure 7:**
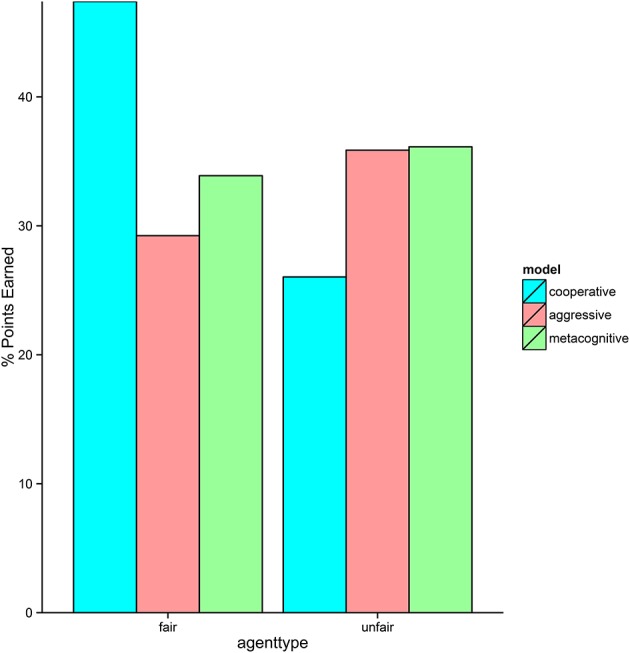
Performance of each version of the model against the two non-cognitive agents.

The three cognitive models performed as expected against the two non-cognitive agents (see Figure 7). The cooperative model performed very well against the fair agent, because it was always honest and not very demanding. However, it was heavily exploited by the unfair agent, resulting in a low score. Conversely, the aggressive model performed very well against the unfair agent because it used final offers liberally. It did not however, do well against the fair agent because it frequently exaggerated its own MNS, causing the fair agent to retaliate with higher bids. The performance of the metacognitive model was more robust, scoring well against both agents. It played just as well as the aggressive model against the unfair agent, but did not play quite as well as the cooperative model against the fair agent. This is most likely due to the fact that the model is uncertain early on about the cooperativeness of its opponent, and therefore is sometimes more aggressive than necessary. On some runs, the model learns that the fair agent is cooperative before it is too late. However, on other runs early aggression escalates into further aggression from both sides, rendering cooperation impossible.

### Quality of fit

**Figure 8:**
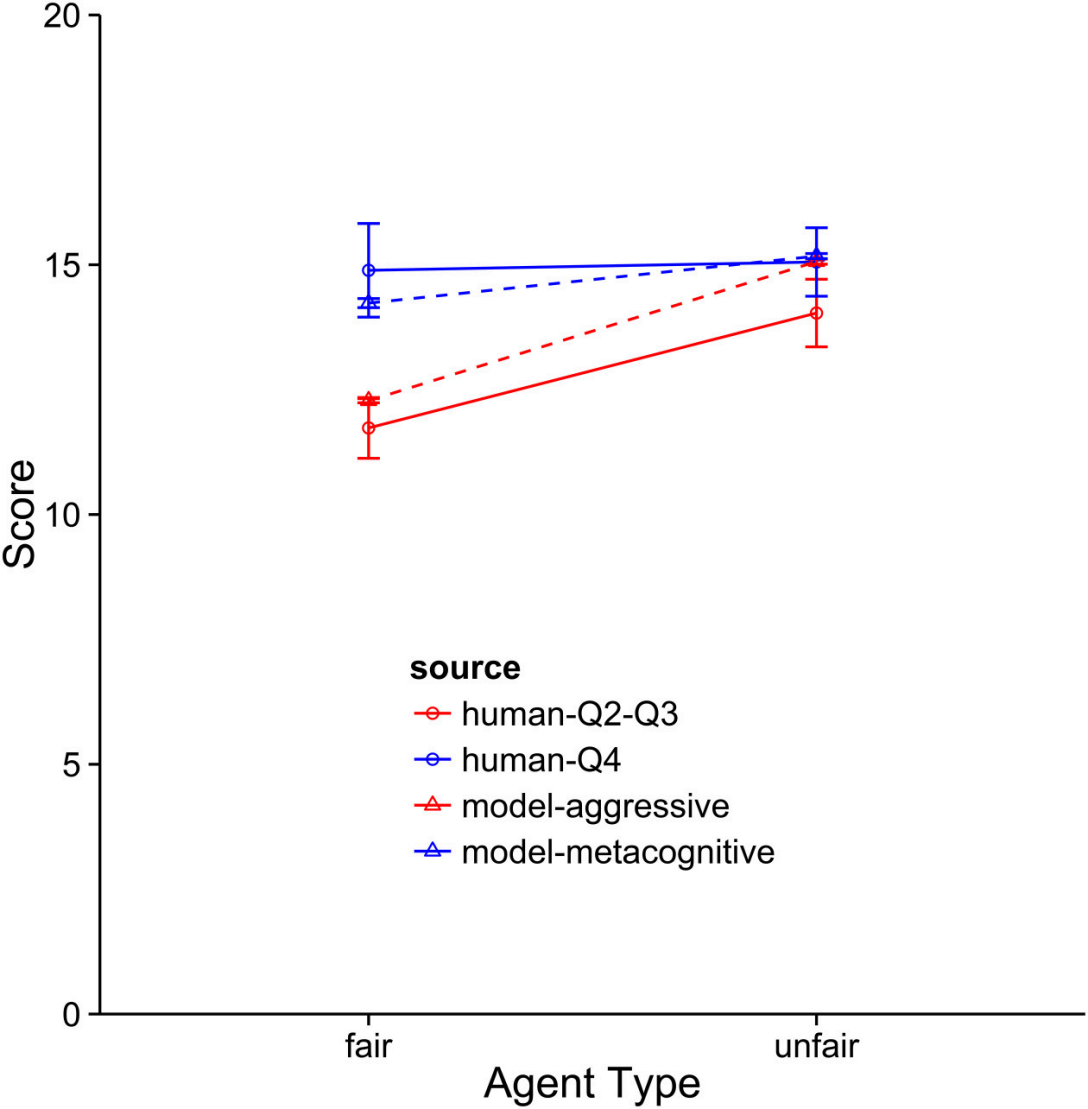
Comparison of the aggressive and metacognitive models to human data with respect to negotiation score. The Y-axis represents raw score per block. Separate lines represent different groups of humans or model types.

**Figure 9:**
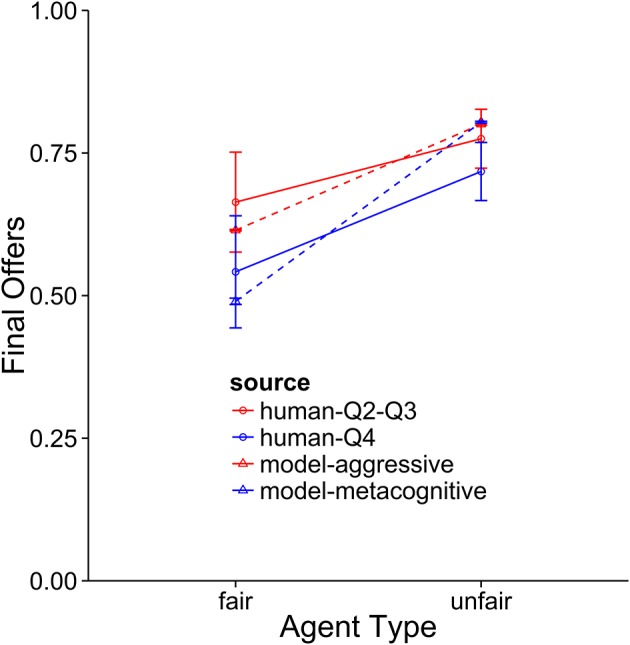
A comparison of the aggressive and metacognitive models to human data with respect to final offer usage. Separate lines represent different groups of humans or model types.

**Figure 10:**
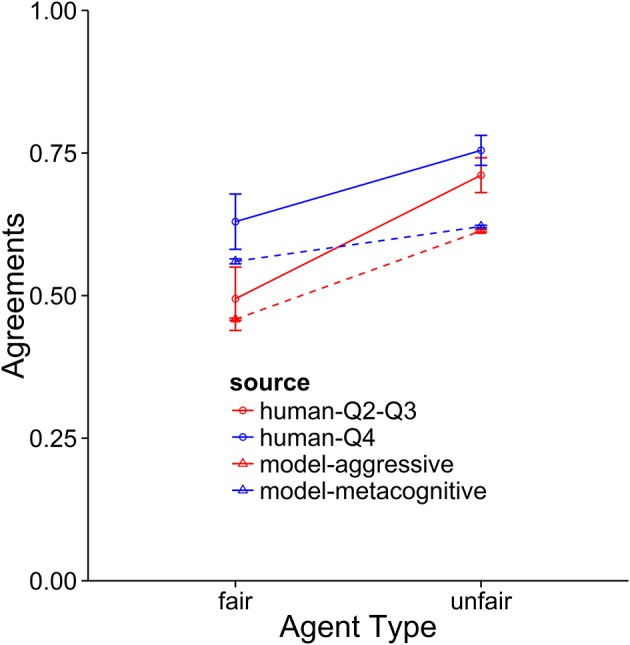
A comparison of the aggressive and metacognitive models to human data with respect to agreements reached. Separate lines represent groups of humans or model types.

The metacognitive model provides the best description of the Q4 subjects (see Figures 8–10). It fits the data best on all three dependent measures: points, final offers, and agreements. The model scores very similarly to human subjects, coming within 1 standard error against both agents. It also provides a good qualitative fit of the usage of final offers, though it slightly overpredicts the final offer usage against the unfair agent. The model also replicates the trend that participants reach more agreements with the unfair agent than with the fair agent. The aggressive model is a slightly worse fit, and the cooperative model is a very poor fit to the data. Overall, this suggests that the top quartile of participants were effective at identifying and matching the strategy of the opposing agent.

When the performance of the models is compared to the middle 50 percent of subjects, it becomes clear that these subjects are using an aggressive strategy and they are not adapting strategies to suit the opposing agent. This is indicated by the fact that the aggressive model provides the best fit for these subjects. In terms of overall score, the model performs at a very similar level as the subjects. It also provides a very good fit for the final offer data and only slightly underpredicts the rate of agreement when playing against the unfair agent. The metacognitive agent did not fit as well, usually because it overpredicted performance against the fair agent. As with the top subjects, the cooperative model was very far from the participant averages.

The subjects in the bottom quartile appear to be using a weak cooperative strategy. Of the three models, the cooperative model comes closest to fitting these data with respect to final offers and agreements. However, the cooperative model scores far better overall than the bottom subjects. This may be explained by the fact that the bottom subjects were more likely than the other groups to accept the agent's offer (rather than continuing to negotiate until the agent settled). On average, the bottom subjects accepted 6.6 offers throughout the experiment, while all other quartiles combined accepted an average of 0.6 offers. They were also more likely than the other groups to quit a negotiation round (*M*_Q1_ = 5; *M*_Q2−Q4_ = 2.03). This suggests these subjects were playing an “impatient cooperative” strategy, in which they quickly gave in to an opponent's demands or ended the round.

## Discussion

These results suggest that adapting to the strategy of even a simple software agent can be difficult for naïve subjects. In this experiment, participants were required to recognize when an agent was behaving cooperatively or aggressively and counter with the appropriate strategy. However, the majority of subjects failed to do this, instead adopting an aggressive strategy regardless of the agent. These subjects failed to recognize that they should play tough against the unfair agent and soft against the fair agent.

Fitting the data in this experiment is a good initial test of the model, but it is known that people negotiate differently with artificial agents than they do with people. In order to confirm that our model is a reasonable, realistic negotiation partner, we needed to know if the model could perform well against humans. This was the object of Experiment 2.

## Experiment 2

Experiment 1 showed that the metacognitive model provides a good description of how people negotiate against artificial agents. However, it was still not clear whether the model can perform realistically against humans. In Experiment 2, we pitted the metacognitive model against human opponents in a scenario similar to a Turing test. This experiment aimed to answer three questions. First, can the model successfully negotiate with human opponents? Second, does the model play against humans similarly to the way humans play against each other? Third, is the model a believable opponent?

### Method

#### Participants

Thirty-eight people participated in the experiment in exchange for a monetary payment of 10 euros. This group consisted mainly of international students at the University of Groningen. The age and gender distribution of this group was similar to that of the participants of Experiments 1 and 3. All were recruited using ads posted on social media.

#### Design

Participants were divided into two groups: human vs. human (*n* = 20, i.e., 10 dyads) and human vs. model (14 dyads). In the human vs. human condition, participants played the Game of Nines against each other through a chat window interface. In the human vs. model condition, participants played the game against a paid confederate operating the model. The purpose of the confederate was to create the appearance that a human opponent was present and to give the model more realistic response times.

#### Procedure

The experimenter briefed both participants on the rules of the game. One participant was then led to a nearby cubicle. Their counterpart was led to a sound-proof room. The participant was told that their counterpart would either be choosing their own moves or operating a computational model. They were instructed that they should select their actions with the goal to get as many points as possible for themselves, not to try to figure out whether the opponent was a human or model. As in the previous experiment, subjects knew that, on average, MNS values for both parties would be the same.

The experimenter sat in a third room and communicated with the participants via the chat window interface. At the beginning of each round, the experimenter privately sent each participant his/her MNS value for the round. In addition, the experimenter told the participants which player should make the first offer. Players alternated making the first offer with every round. Fourteen total trials were played, using the following MNS values in random order (1,1) (2,2) (3,3) (4,4) (1,3) (3,1) (1,5) (5,1) (3,4) (4,3) (2,6) (6,2) (4,5) (5,4).

Because the model is not designed to generate text or speech (other than a few basic statements), all participants were given a fixed set of statements from which they could select. These included stating a specific offer, declaring a final offer, accepting an opponent's offer, and quitting the round. Participants were asked to always use the same wording when using these moves (e.g., to accept an offer, a player would always type “deal”).

Finally, when all rounds of the negotiation were complete, the participant in the cubicle was given a questionnaire and asked to answer several questions about the counterpart. These included questions about the counterpart's agreeability, likability, and humanity. All questions were answered using a 10-point Likert scale.

#### The model

Pilot testing indicated that a few participants could exploit the model, putting it into a cooperative state when it should have been aggressive. One possible reason for this is that the final offer policy of both cooperative and aggressive strategies was too lenient. Thus, after pilot testing, the instances used in the metacognitive agent were modified (see Appendix A in Supplementary Material). Specifically, the model was given the ability to quit a trial if it appeared that the final agreement was not going to be worthwhile. The model would quit if the value of the model's current offer was too low. Furthermore, the model quits sooner while in the aggressive state than it does in the cooperative state.

Confederates interacted with the model through a GUI. Using the GUI, confederates could input the offer made by the other player and whether it was a final offer. In turn, the agent indicated the move that the confederate should make to the participant.

### Results

Three dyads in the human vs. model condition were excluded because the human confederate made errors when operating the model. This resulted in 11 human vs. model dyads and 10 human vs. human dyads remaining in the final dataset.

Three major results emerge from this experiment. First, the model was able to successfully negotiate with human partners, achieving more points from the negotiation than its partners (although human-model dyads reached fewer agreements than human-human dyads). Second, the model achieved similar levels of performance in terms of trials quit and final offers made. Finally, the model achieved a degree of believability, as humanity ratings did not differ between the human players and the confederates operating the model.

In terms of overall score, the model performed just as well as the human counterparts (see Table 5). However, it played a bit more aggressively than humans do. Examining the other dependent variables suggests that the model otherwise plays similarly to humans. The model's use of final offers and quits is not significantly different from those of the human players (*p* > 0.1), but there is a trend such that the model quits more often and uses final offers more often than human players do. As a result, the model's partners scored lower on average than those who played against humans [*t*_(16.778)_ = −3.70, *p* < 0.002]. In addition, the model chose to use a cooperative strategy on only 32% (SE = 8%) of the trials. This means that on the majority of trials, the model believed the player was acting aggressively and responded accordingly.

**Table 5:** Comparison of model performance to human performance in Experiment 2.

**Player 1**	**Participant score**	**Human/model partner**	**Player 1 final offers**	**Partner final offers**	**Player 1 quits**	**Partner quits**
Model	9.7 (2.8)^**^	13.9 (3.4)	5.7 (2.5)	5.1 (2.2)	4.3 (1.9)	4.5 (1.9)
Human	15.3 (3.7)	13.8 (4.1)	4.4 (1.5)	4.4 (1.5)	3 (1.3)	3 (1)

Values in parentheses are standard deviations. Each round contained 14 trials, making the maximum number of quits and final offers 14. “Counterpart” refers to the human participant playing the game from outside the sound proof chamber.

** *Model performance is significantly different from human performance at p < 0.01*.

The results of the humanity survey were encouraging. On average, players gave the model a humanity rating of 5.82 and gave the human players a rating of 5.6 (|*t*| < 1). This result suggests that, over the course of our short negotiation game, the model's behavior was plausible to participants.

### Discussion

Experiment 2 is a successful test case for the metacognitive model. Our results indicate that the model performs just as well against human players as humans do. Further, its play style is similar to humans with the exception that it is slightly more aggressive. Finally, the model was able to pass our simple Turing Test, achieving similar humanity ratings as real human participants.

It should be noted, however, that the model does not appear to be a perfect fit. It seems that the model plays more aggressively overall than humans do. The model quit more often and used final offers more often than the human vs. human players did. It also seems to have caused similar behavior in its partners. Most importantly, the model allowed its partners to have far fewer points than the human players did. The model chose the cooperative strategy on only 33% of all steps. It is not the case that the model simply played aggressively against the players regardless of the players' behavior. The better performing subjects were better able to encourage the model to cooperate. Against the top five scoring subjects, the model chose the cooperative strategy 42% of the time.

## Experiment 3

Experiment 2 showed that our metacognitive model negotiates effectively and believably against human players. In Experiment 3, we aimed to determine whether practicing with our metacognitive model could improve theory of mind skills. This time, participants interacted directly with the model (without a human intermediary). We hypothesized that playing against an adaptive agent would better help participants learn theory of mind than the simple agents of Experiment 1.

Effective use of theory of mind requires that participants notice that the model has beliefs about their behavior and adjusts its own behavior accordingly. This may be difficult for participants to recognize in a complex task like negotiation. More difficult still is understanding what these beliefs are and how they can manipulate these beliefs. Therefore, to facilitate learning, we included a condition in which participants received explicit feedback about the model's beliefs. After every bid, these participants were notified whether the model believed their behavior was cooperative or aggressive and whether the model was playing cooperatively or aggressively.

### Participants

43 people (13 males, age *M* = 21.52, *SD* = 2.07) participated in the experiment in exchange for 10 euros. This group consisted mainly of international students at the University of Groningen. All were recruited using ads posted on social media.

### Design and procedure

The experiment consisted of two phases: a training phase and a test phase. In the training phase, participants were assigned to either the feedback condition or the no-feedback condition. Participants in the feedback condition received information about the model's beliefs (described in greater detail in the Cognitive Agent section). In the test phase, all participants received no feedback.

In both the training phase and the test phase, the participants played the Game of Nines against the metacognitive agent for 3 blocks of 12 trials. As before, a trial consisted of each player being assigned an MNS value and negotiating until agreement or quitting. The following pairs of MNS values were used: (1,1) (2,2) (3,3) (4,4) (1,3) (3,1) (1,5) (5,1) (2,6) (6,2) (4,5) (5,4). Each pair was used once per block in random order.

### Metacognitive agent

The metacognitive agent contained the same instances (and therefore behaved the same way) as the agent in Experiment 2. Participants interacted with the agent using a graphical interface. Like the interface for the non-cognitive agents, this interface contained buttons for the player to indicate their bid and to declare a final offer. Moreover, the player could choose to accept the agent's offer or quit the round on any turn. At the end of a round, the player was asked to press the “Next Round” button to begin a new round. Regardless of feedback condition, all players could see the number of points they had earned in a given block. However, the players received no information about the model's score.

#### Theory of mind feedback

**Figure 11:**
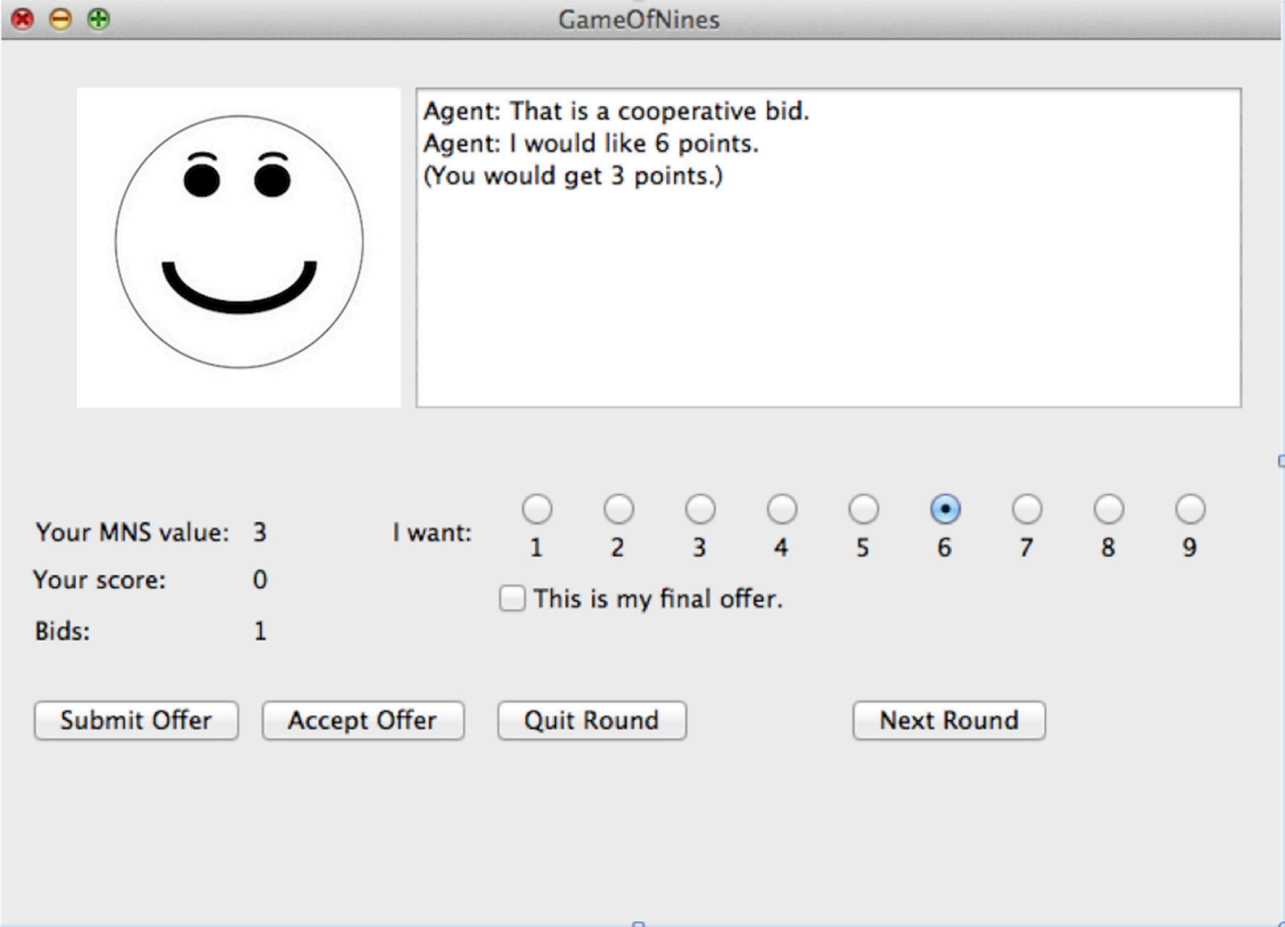
A screenshot from the model in the feedback condition. In the no-feedback condition, no face was shown on the left side and statements evaluating the player's move (e.g., “That is a cooperative bid.”) did not appear.

Participants in the feedback condition received information about the model's current strategy and the model's evaluation of the player's last move. If the model was using a cooperative strategy, a smiley face was shown in the graphical interface. But if the model was using an aggressive strategy, a frowning face was displayed instead. In addition, the model displayed a message to the player showing whether the model thought the player's move was cooperative, aggressive or neutral. In the no-feedback condition, no face appeared in the interface and no evaluation messages were displayed. See Figure 11 for a screenshot of the display.

### Results

Two participants received a negative total score for the experiment, indicating that they either ignored or failed to understand the instruction to not accept any deals of negative value. Therefore, these two participants were removed from all analyses. In all of the following analyses, we used Linear Mixed Effects (LME) models (Bates et al., 2014; Kuznetsova et al., 2015) with subjects as a random effect.

**Figure 12:**
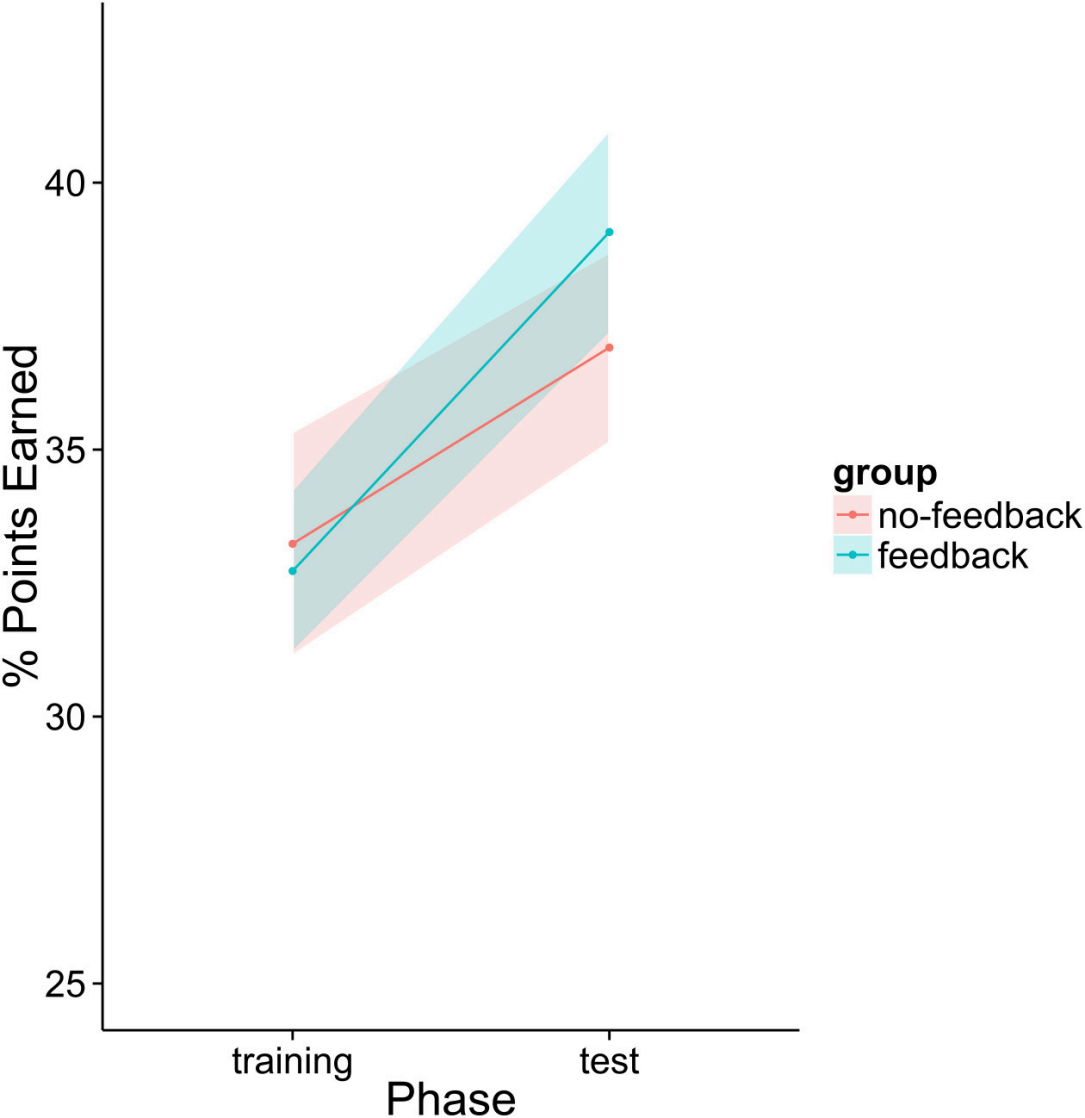
Percentage of points earned by the player as a function of group and experiment phase. Fifty percent represents an even split between the human player and the model.

First, we analyzed the participants' performance data as a function of phase and feedback group. The main effects of both variables as well as their interaction term were entered into the model. Participants had significantly higher scores in the second phase than in the first phase regardless of feedback condition [*t*_(39)_ = 2.305, *p* = 0.0266]. There was a numerical trend such that participants in the feedback condition performed better overall (*M* = 35.9% points earned) than those in the no-feedback condition (*M* = 35.1%) and participants in the feedback condition appear to have improved more from phase 1 to phase 2 than those in the no-feedback group (see Figure 12). However, neither of these trends was statistically significant (*p* > 0.1).

**Figure 13:**
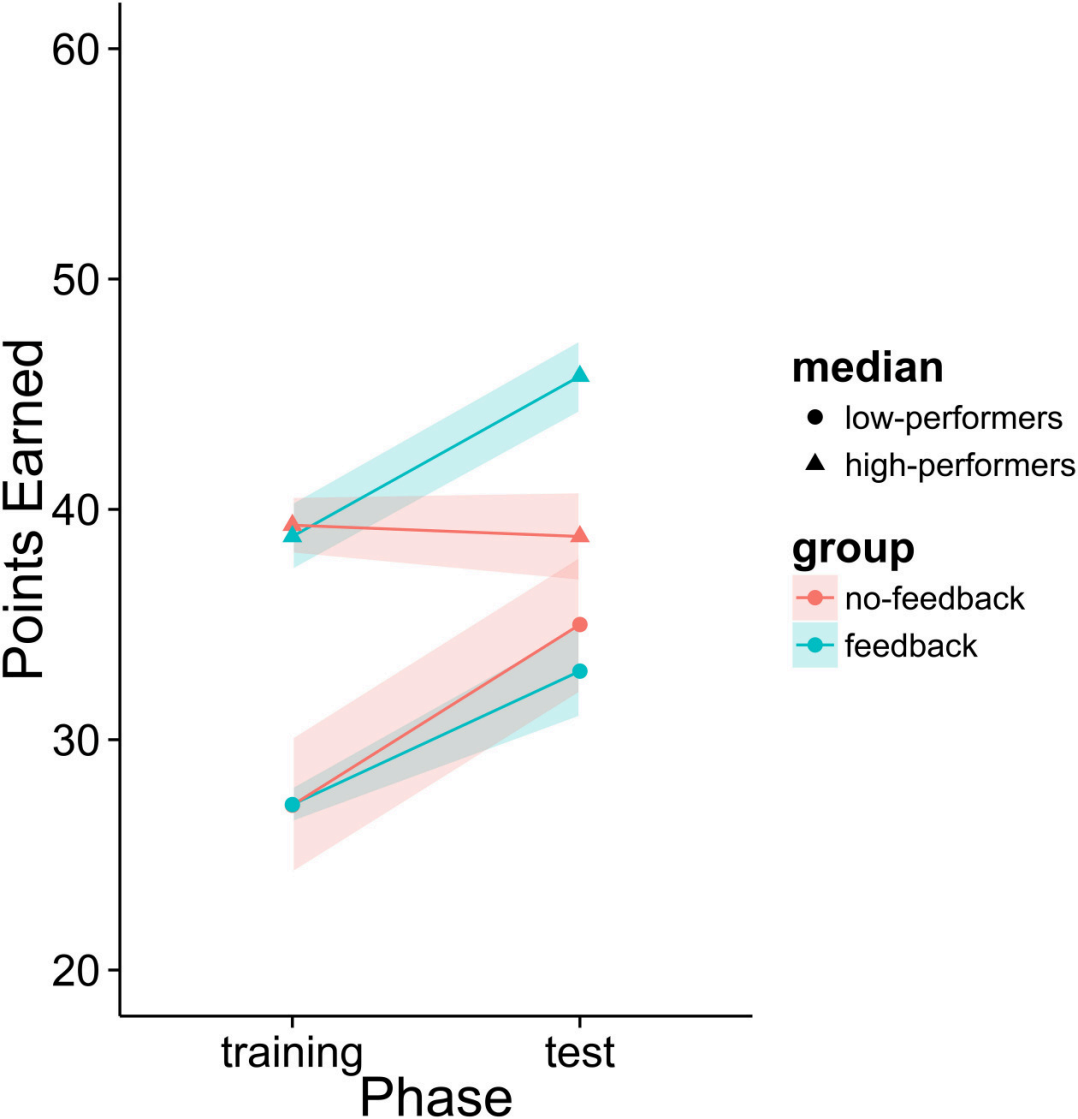
Percentage of points earned after dividing participants into high and low performance groups.

The initial analysis supported our hypothesis that training with the metacognitive agent would yield measurable improvements in negotiation scores. However, it did not support our hypothesis that providing theory of mind feedback would enhance these gains (see Table 6 and Figure 12). Yet, visual inspection of the individual performance curves suggested that subjects who performed better in the training phase showed stronger improvements in the test phase. To investigate this possibility, we performed a median split based on subject performance in the training phase. Participants who scored below the median were placed into the low-median group and those above the median were in the high-median group. We then submitted these data to a LME model with feedback group, phase, and median (as well as their interactions) as fixed effects and subjects as a random effect. This analysis revealed a three-way interaction between feedback, phase and median (see Table 7 and Figure 13). Those in the low-performance group showed roughly the same amount of improvement in the test phase, regardless of feedback. However, those in the high-median group showed improvement only when receiving feedback.

**Table 6:** LME results by feedback group and by experiment phase.

**Fixed factor (reference group)**	**Estimate**	**SE**	***df***	***t***	***p***
Feedback group (no feedback)	−0.519	2.639	55.9	0.197	0.845
Phase (1)	3.750	1.627	39	2.305	0.0266
Phase × group	2.726	2.273	39	1.199	0.237

**Table 7:** LME results after splitting data into high and low performance groups.

**Fixed factor (reference group)**	**Estimate**	**SE**	***df***	***t***	***p***
Feedback group (no feedback)	0.027	2.82	61.52	0.01	0.992
Phase (1)	8.0	2.14	37	3.376	< 0.001
Median (low)	12.4	2.88	61.52	4.293	< 0.001
Group × Phase	−2.09	2.96	37	0.707	0.484
Group × Median	−0.527	4.04	61.5	0.131	0.897
Phase × Median	−8.5	3.03	37	2.807	0.00793
Group × Phase × Median	9.69	4.23	37	2.289	0.0278

In order to examine differences in learning rates between experiments, we analyzed data from the training phase alone. The training phase contained the same number of trials as Experiment 1. To determine if detectable learning occurred in the training phase of the experiment, we fit a linear mixed-effects model to the three blocks of the training phase (Table 8). This analysis indicates significant improvement in terms of negotiation outcomes after the same amount of practice as in Experiment 1 (whereas no significant effect emerged in Experiment 1). Though preliminary, this result suggests a benefit to training with cognitive agents as opposed to non-cognitive agents.

**Table 8:** LME analysis on points earned in the training phase of Experiment 3.

	**Estimate**	**SE**	***df***	***t***	***p***
Block	1.93	0.46	80	4.143	< 0.05^*^
Feedback group (no feedback)	0.61	1.56	119	4.14	0.70
Block × Feedback group	0.22	0.64	80	0.34	0.73

* *Means statistically significant at α = 0.05*.

### Discussion

Two important results emerge from Experiment 3. First, students training against our metacognitive agent showed significant improvement in negotiation scores whereas the students that played against the fixed-strategy agents in Experiment 1 did not. This suggests that playing against an agent with theory of mind may be more helpful than playing against one without, at least for relatively short training periods. The second key finding is that additional feedback about the model's metacognitive state helped only participants who were already performing well. Low performers benefited just as much with or without feedback. This suggests that acquiring theory of mind is cognitively demanding, and students cannot make use of the feedback if they are still mastering other, more basic elements of the task (e.g., how and when to make bids, basic tactics, operating the interface, etc.). We suspect that both of the low performing groups were learning basic aspects of the task and the low-performing feedback group likely either ignored the theory of mind feedback or did not know how to make use of it.

## General discussion

Training in interpersonal skills like negotiation can be expensive and time-consuming. Training with artificial agents can help by providing a consistent training experience that does not require a coach or a training partner. Cognitive architectures, like ACT-R, can help to produce artificial agents that behave more similarly to humans than traditional approaches. Here we present an example artificial agent based on a cognitive model built in ACT-R. Experiment 1 showed that this agent replicates common strategies used by human participants. Experiment 2 showed that this agent can interact with humans in a believable way and that human-agent dyads can perform almost as well as human-human dyads. Finally, Experiment 3 showed that participants can improve their performance in the Game of Nines by practicing with the agent. Overall, cognitive architectures may be valuable assets in the creation of artificial training partners for interpersonal skills.

We believe that training with metacognitive agents is potentially a useful training technique. Training with an artificial agent has been shown to be effective in improving negotiation skill (Lin et al., 2014; de Weerd et al., 2015a). A cognitive agent could demonstrate human-like cooperative and aggressive strategies, training the student to better recognize the two. Additionally, practicing against a cognitive agent could allow a participant to try out cooperative and aggressive strategies, and thus better learn when to use them. More generally speaking, we believe cognitive agents have great potential as training partners in a variety of domains.

There are many existing artificial agents that allow people to practice negotiation (Carneiro et al., 2012; Lin et al., 2014; Cao et al., 2015). However, an important feature of our agent for education is cognitive plausibility. Human behavior often deviates from normative rationality, so training with an agent that exhibits realistic biases and cognitive limitation can provide exposure to situations during training that would not occur with a normative agent (e.g., mis-remembering prior interactions, hasty generalizations). It remains an open question which types of cognitive and behavioral phenomena are most important to include in training. However, answering this question could improve training outcomes by maximizing focus on those situations a student is most likely to see.

Our model is built on the assumption that people use theory-of-mind (at least to a degree) when engaging in social interactions and games. Existing cognitive models of trust have been effective in modeling performance in coordination games (e.g., the Prisoner's Dilemma and Chicken: Juvina et al., 2014; Collins et al., 2015). These models assume that trust develops according to a utility function. When cooperation is rewarded, the model will trust the opponent more. However, a utility-learning approach may be difficult to employ in situations where the payoff is delayed or uncertain. In our model, the development of trust occurs primarily in declarative memory. People compare their counterpart's behavior to examples of cooperative and aggressive behavior they have encountered in the past. Using this process, they can determine whether the person is cooperative or aggressive. It is possible that the development of trust in real-world settings involves a combination of these two types of processes.

The present work only scratches the surface of the complexities and nuances of theory-of-mind reasoning. The present model attempts to infer only its counterpart's strategic stance. However, theory of mind also includes a variety of complex reasoning processes, including reasoning about a counterpart's beliefs and about a counterpart's beliefs about the agent's beliefs (de Weerd et al., 2015b).

The agent may also provide insights into why theory of mind sometimes fails. In negotiation, people usually do not have access to specific information about the circumstances of their counterpart. In the present experiments, the model did not have access to the opponent's current MNS value, so it substituted a noisy average based on its own past MNS values. This sometimes led to errors (e.g., believing an opponent's MNS was 2 when it was really 4). In real world negotiations, people may have very little information about their counterparts' reserve prices, potentially leading firmness to be misconstrued as greed (and vice versa). Thus, our approach may potentially be useful when searching for circumstances that may lead to mistrust and misunderstandings.

Our model provides an account of how people use instance-based reasoning to infer the intentions of others. However, it remains an open question how people acquire new instances and how they classify those instances as cooperative or aggressive. One way people may do this is by comparing newly observed instances to stored instances and rating their overall similarity to cooperative and aggressive instances. Alternatively, people may associate instances with particular people. They may then evaluate the strategies of new people by comparing them to people they have observed in the past.

To ensure that the behavior of the model was plausible, its instances were written by the authors, not by a learning algorithm. The assumption here is that this set of instances represents one possible learning history that could produce the observed behavior. There are many different possible sets of instances that could be used for such a model, but these different sets would potentially result in different patterns of behavior than that of the present model. Most other possible sets of instances are likely to produce erratic or ineffective behavior in our task, but we acknowledge that other sets of instances exist that may produce plausible behavior. Nevertheless, the use of instances to infer the mental state of the opponent proved to be an effective strategy for producing a model that performs well and is believable to human players.

An important type of negotiation not addressed by the current model is integrative negotiation. Integrative negotiation refers to the process through which negotiators work together to find opportunities to improve joint value. By contrast, distributive negotiation refers to the process of deciding how to divide the available value among the negotiators. In other words, integrative negotiation concerns how to “grow the pie,” and distributive negotiation concerns how to “divide the pie” (Kersten, 2001). In the Game of Nines, the only integrative component is the fact that if players fail to reach an agreement, they both lose an opportunity to gain points. In real-world negotiations, integrative negotiation involves exchanging information about preferences (Bartos, 1995; Kersten, 2001). We believe our agent will still be helpful for students in these negotiations because the central skill trained by our agent, theory of mind, is also important (perhaps even more so) in integrative negotiation (Hindriks and Tykhonov, 2008; de Weerd et al., 2013). It is possible that the benefits of training negotiation in this simple scenario could transfer to more complicated scenarios, but additional research is necessary to confirm this.

## Conclusion

In negotiations, the behavior of one's counterpart is often ambiguous. Successful negotiators must infer the intentions of their opponents and adjust their own behavior accordingly. Misinterpreting and failing to reciprocate a cooperative gesture could turn an otherwise cooperative opponent into an aggressive one. However, cooperating with an aggressive opponent may result in exploitation. Our model provides an example of how artificial teammates could be built to train people to adapt to different strategy types in social tasks.

## Author notes

CS is now in the Cognitive Models and Agents Branch of the 711th Human Performance Wing at the U.S. Air Force Research Laboratory. The opinions expressed herein are solely those of the authors and do not necessarily represent the opinions of the United States Government, the U.S. Department of Defense, the U.S. Air Force, or any of their subsidiaries, or employees. This work was not financed by the above organizations.

## Ethics statement

This study was carried out in accordance with the recommendations of the Ethics Committee Psychology of the University of Groningen with written informed consent from all subjects. All subjects gave written informed consent in accordance with the Declaration of Helsinki. The protocol was approved by the Ethics Committee Psychology.

## Author contributions

CS: Wrote the manuscript, provided guidance and support in running Experiments 2 and 3, wrote the code for the models and agent software, and conducted follow-up data analyses; JD and EG: Designed, ran, and wrote up Experiment 1; TR and JT: Designed, ran, and wrote up Experiments 2 and 3; FC: Assisted in planning Experiments 2 and 3 and made significant edits to the manuscript; NT: Conceived of the line of research, provided significant theoretical and technical guidance to the other authors regarding the design of all three experiments and the development of the accompanying agents, and made substantial edits to the manuscript.

### Conflict of interest statement

The authors declare that the research was conducted in the absence of any commercial or financial relationships that could be construed as a potential conflict of interest.
