# Supplementary material for: Using Cognitive Agents to Train Negotiation Skills
Source: Front Psychol. 2018 Feb 19;9:154. doi: 10.3389/fpsyg.2018.00154 (PMC5835330; doi:10.3389/fpsyg.2018.00154)
Supplement: Supplementary file 1 [file DataSheet1.docx]

**Appendix A: Instances for models in Experiment 1 and Experiment 2**

Table A.1. List of instances for the models in Experiment 1.

| Move-Type | Strategy | Model-MNS | Bid-Diff | Opponent-move | My-move |
| --- | --- | --- | --- | --- | --- |
| Claim | Cooperative | 1 | N/A | N/A | MNS+0 |
| Bid | Cooperative | N/A | 3 | -2 | -2 |
| Bid | Cooperative | N/A | 2 | -2 | -2 |
| Bid | Cooperative | N/A | 2 | -1 | -1 |
| Bid | Cooperative | N/A | 1 | -1 | -1 |
| Bid | Cooperative | N/A | 3 | 0 | -1 |
| Decision | Cooperative | N/A | 1 | -1 (final) | Reject |
| Decision | Cooperative | N/A | 2 | -1 (final) | Reject |
| Opening | Cooperative | 1 | N/A | 6 (opening) | 5 |
| Opening | Cooperative | 4 | N/A | 6 (opening) | 6 |
| Opening | Cooperative | 1 | N/A | 5 (opening) | 5 |
| Opening | Cooperative | 4 | N/A | 8 (opening) | 7 |
| Claim | Aggressive | N/A | N/A | N/A | MNS+1 |
| Decision | Aggressive | N/A | 1 | -1 (final) | Reject |
| Decision | Aggressive | N/A | 2 | -1 (final) | Accept |
| Final-offer | Aggressive | N/A | 2 | 0 | -1 (final) |
| Final-offer | Aggressive | N/A | 3 | 0 | -2 (final) |
| Opening | Aggressive | 1 | N/A | 8 (opening) | 9 |
| Opening | Aggressive | 4 | N/A | 6 (opening) | 7 |
| Opening | Aggressive | 1 | N/A | 7 (opening) | 8 |
| Opening | Aggressive | 4 | N/A | 7 (opening) | 8 |
| Bid | Neutral | N/A | 2 | -2 | -1 |
| Bid | Neutral | N/A | 3 | -1 | -1 |
| Bid | Neutral | N/A | 1 | -1 | 0 |
| Bid | Neutral | N/A | 5 | -1 | -1 |
| Bid | Neutral | N/A | 5 | 0 | -1 |
| Final-offer | Neutral | N/A | 1 | 0 | 0 |
| Decision | Neutral | N/A | 1 | -1 (final) | Accept |
| Decision | Neutral | N/A | 0 | -1 (final) | Reject |
| Opening | Neutral | 3 | N/A | 7 (opening) | 7 |

*Note.* “Claim” instances refer to the model’s decision whether to lie about its MNS value. “Opening” instances determine the model’s first offer in a round. “Bid” instances determine whether the model should concede or insist. “Decision” instances determine how to respond to a final offer. “Final-offer” instances tell the model to issue a final offer. The “Bid-diff” column refers to the difference between the model’s current bid and its MNS value. Opponent-move codes the move the opponent has just made. “My-move” represents the model’s move if the instance is retrieved. A positive number in either of these columns indicates the absolute value of the opponent’s (or model’s) current bid. Negative numbers indicate that the bid has been reduced by the indicated amount from the previous bid.

Table A.2. Instances for the metacognitive model in Experiment 2

| Move-Type | Strategy | Model-MNS | Bid-Diff | Opponent-move | My-move |
| --- | --- | --- | --- | --- | --- |
| Bid | Cooperative | N/A | 3 | -2 | -2 |
| Bid | Cooperative | N/A | 2 | -2 | -2 |
| Bid | Cooperative | N/A | 2 | -1 | -1 |
| Bid | Cooperative | N/A | 1 | -1 | -1 |
| Bid | Cooperative | N/A | 3 | 0 | -1 |
| Bid | Cooperative | N/A | 1 | 0 | 0 |
| Decision | Cooperative | N/A | 1 | -1 (final) | Reject |
| Decision | Cooperative | N/A | 2 | -1 (final) | Reject |
| Opening | Cooperative | 1 | N/A | 6 (opening) | 5 |
| Opening | Cooperative | 4 | N/A | 6 (opening) | 6 |
| Opening | Cooperative | 1 | N/A | 5 (opening) | 5 |
| Opening | Cooperative | 4 | N/A | 8 (opening) | 7 |
| Bid | Aggressive | N/A | 3 | 1 | 0 |
| Bid | Aggressive | N/A | 2 | 1 | 0 |
| Decision | Aggressive | N/A | 1 | -1 (final) | Reject |
| Decision | Aggressive | N/A | 2 | -1 (final) | Accept |
| Final-offer | Aggressive | N/A | 2 | 0 | 0 (final) |
| Final-offer | Aggressive | N/A | 3 | 0 | -1(final) |
| Opening | Aggressive | 1 | N/A | 8 (opening) | 9 |
| Opening | Aggressive | 4 | N/A | 6 (opening) | 7 |
| Opening | Aggressive | 1 | N/A | 7 (opening) | 8 |
| Opening | Aggressive | 4 | N/A | 7 (opening) | 8 |
| Quit | Aggressive | N/A | 2 | 1 | quit |
| Bid | Neutral | N/A | 2 | -2 | -1 |
| Bid | Neutral | N/A | 3 | -1 | -1 |
| Bid | Neutral | N/A | 1 | -1 | 0 |
| Bid | Neutral | N/A | 3 | 0 | 0 |
| Bid | Neutral | N/A | 2 | 0 | 0 |
| Final-offer | Neutral | N/A | 1 | 0 | 0 |
| Final-offer | Neutral | N/A | 2 | 0 | -1 |
| Decision | Neutral | N/A | 1 | -1 (final) | Accept |
| Decision | Neutral | N/A | 0 | -1 (final) | Reject |
| Opening | Neutral | 3 | N/A | 7 (opening) | 7 |
| Quit | Neutral | 1 | 0 | 0 | quit |

**Appendix B: Implementation details of the fair and unfair agents.**

**Computing Target Offers**

The fair agent computes a target offer that splits the points as evenly as possible between the two players given their MNS values. Because the agent does not have access to the player’s true MNS value it uses the player’s claimed MNS value as the basis for the decision and adjusts it if it detects dishonesty. This adjustment is performed by the following formula.

$$aMNS=round(cMNS-A*\frac{diffMNS}{se(MNSpa)})$$

Where:

aMNS: adjusted MNS

A: a constant representing the weighting of the trust value. For the fair agent, this is set to 1.

cMNSp: The player’s claimed MNS value

diffMNS: The difference between the means of the agent’s MNS values and the claimed values of the player

MNSpa: A combined list of the MNS values of the agent and the player’s claimed values

se(MNSpa): The standard error in the MNS values, which is an indication of uncertainty that decreases with more experience. In other words, the agent generates an estimate of how much its opponent is lying, then it adjusts the opponent’s claim by that amount. As a result, the agent will ask for more points when the player lies about his or her MNS.

The unfair agent uses the same formula as the fair agent to calculate a target offer with two exceptions. First, it will use its claimed MNS value (see next section) rather than its actual value. This results in the agent making more aggressive offers on average than the fair agent. Second, it does not weigh its opponent’s lies as heavily (the constant, A, is set to .5 rather than 1).

MNS Claims

The fair agent always honestly reports its MNS value. The unfair agent, by contrast, exaggerates its MNS value using the following formula. This formula ensures that the agent will exaggerate less as its MNS approaches 4, preventing absurdly high MNS claims.

$$cMNS=MNS+round(\frac{4}{MNS})$$

Both agents will accept an offer only if it is at least as good as their computed “fair offer.” If the player asks for more than this, the agent will propose a counter offer using the following formula:

$$\text{offer}_{agent}=\text{fair}_{agent}+(\text{offer}_{player}+ \text{fair}_{player})$$

Where *fair* denotes the agent’s calculation of how many points the agent and the player should receive respectively. In other words, the agent will make an offer that is just as high above its own fair value as the opponent’s offer is below the agent’s fair value.
